# Recent Insights into Cellular and Molecular Mechanisms of Defective Angiogenesis in Systemic Sclerosis

**DOI:** 10.3390/biomedicines12061331

**Published:** 2024-06-14

**Authors:** Eloisa Romano, Irene Rosa, Bianca Saveria Fioretto, Mirko Manetti

**Affiliations:** 1 Section of Internal Medicine, Department of Experimental and Clinical Medicine, University of Florence, Largo Brambilla 3, 50134 Florence, Italy; eloisa.romano@unifi.it; 2 Section of Anatomy and Histology, Department of Experimental and Clinical Medicine, University of Florence, Largo Brambilla 3, 50134 Florence, Italy; irene.rosa@unifi.it (I.R.); biancasaveria.fioretto@unifi.it (B.S.F.); 3 Imaging Platform, Department of Experimental and Clinical Medicine, University of Florence, Largo Brambilla 3, 50134 Florence, Italy

**Keywords:** systemic sclerosis, scleroderma, angiogenesis, endothelial cells, endothelial-to-mesenchymal transition, vasculopathy

## Abstract

In systemic sclerosis (SSc, or scleroderma), defective angiogenesis, clinically manifesting with abnormal capillary architecture and severe capillary reduction, represents a hallmark of early-stage disease, usually preceding the onset of tissue fibrosis, and is caused by several cellular and molecular mechanisms affecting microvascular endothelial cells with different outcomes. Indeed, once damaged, endothelial cells can be dysfunctionally activated, thus becoming unable to undergo angiogenesis and promoting perivascular inflammation. They can also undergo apoptosis, transdifferentiate into profibrotic myofibroblasts, or acquire a senescence-associated secretory phenotype characterized by the release of exosomes and several profibrotic and proinflammatory mediators. In this narrative review, we aimed to give a comprehensive overview of recent studies dealing with the cellular and molecular mechanisms underlying SSc defective angiogenesis and the related endothelial cell dysfunctions, mainly the endothelial-to-mesenchymal transition process. We also discussed potential novel vascular treatment strategies able to restore the angiogenic process and reduce the endothelial-to-mesenchymal transition in this complex disease.

## 1. Introduction

Angiogenesis, the complex multi-step process of new vessel formation from pre-existing ones, is finely regulated by pro- and anti-angiogenic factors that are in dynamic equilibrium in physiological conditions, but out of balance in pathologies such as cancer, rheumatoid arthritis, and systemic sclerosis (SSc) [1,2,3,4,5,6]. Among the different angiogenic variants, sprouting angiogenesis, during which endothelial cells (ECs) sprout from a capillary to form a novel capillary network, is commonly considered the dominant one in adult subjects [1,5,7]. In particular, endothelial sprouting, which may occur after exposure to hypoxia, injury, or angiogenic growth factors like the vascular endothelial growth factor (VEGF), starts with a raise in vascular permeability and consequent extravasation of plasma proteins acting as a provisional scaffold for ECs [1,7]. Once activated, specific ECs known as “tip” and “stalk” cells proliferate and migrate into the surrounding tissue by producing and secreting matrix metalloproteinases (MMPs) that degrade the vascular basement membrane and the perivascular extracellular matrix (ECM), thus forming primary sprouts [1,7]. Such newly formed vessels are initially immature and leaky, but through the subsequent interaction with other cell types such as pericytes, they undergo remodeling and stabilization, ultimately maturing into complete and functional tube-like structures [1,7]. Another variant of angiogenesis is represented by intussusceptive or splitting angiogenesis, which is characterized by the formation of an intraluminal pillar that, longitudinally splitting the vascular lumen into two, generates smaller vessels, thus expanding the vascular bed. Not depending on EC proliferation, intussusception is a rapid vascular remodeling process that may occur within hours or even minutes [1,7]. An additional recently described form of angiogenesis, also called inverse intussusception, is coalescent angiogenesis, during which blood vessels merge into larger structures, thus increasing the microcirculation efficiency [1,7].

SSc, also referred to as scleroderma, is a severe connective tissue disorder in which defective angiogenesis, manifesting with abnormal peripheral capillary architecture and progressive and severe capillary reduction, not followed by a compensatory sprouting process, represents a hallmark of early-stage disease, usually preceding the onset of tissue fibrosis [8,9,10,11]. Characteristic microvascular changes can be assessed in the nailfold area of SSc patients by nailfold videocapillaroscopy (NVC) and include giant capillaries, ramified capillaries, hemorrhages, and avascular areas [2,6,11]. In particular, the SSc capillaroscopic patterns can be classified into (i) “early NVC”, characterized by microhemorrhages and rare giant capillaries, without an evident capillary loss; (ii) “active NVC”, with frequent giant capillaries and microhemorrhages, moderate capillary loss, and slight disorganization of the capillary architecture; and (iii) “late NVC”, featuring extensive avascular areas and the absence of microhemorrhages [2,6,11]. From a clinical point of view, SSc patients show a wide spectrum of peripheral vascular dysfunctions, with Raynaud’s phenomenon being the earliest manifestation and leading to constant ischemia that further develops into digital ulcers (DUs) or, in extreme cases, gangrene [8,9,10,11]. Pulmonary arterial hypertension (PAH) and scleroderma renal crisis represent two other important vasculopathy manifestations that, although silent in the early disease stage, can be potentially lethal [8,9,10,11].

## 2. Objective and Methods

In this narrative review, we aimed to give a comprehensive overview of recent studies dealing with the cellular and molecular mechanisms underlying SSc defective angiogenesis and related endothelial cell dysfunctions, mainly the endothelial-to-mesenchymal transition (EndoMT). We also discussed potential novel vascular treatment strategies able to restore the angiogenic process and reduce the EndoMT in this complex disease. As a methodology, an electronic search was conducted across the online databases Web of Science, Scopus, and PubMed using the relevant medical subject headings (MeSH) and keywords including “angiogenesis”, “endothelial cells”, “endothelial-to-mesenchymal transition”, “vascular”, “systemic sclerosis”, and “scleroderma”. These MeSH terms and keywords were combined with “AND/OR” Boolean operators. The search was limited to English language articles (both original and review articles) published in the 2020–2024 period. Some reference lists of the selected articles were further reviewed to identify relevant studies.

## 3. Endothelial Cell Dysfunction and Impaired Angiogenesis in SSc

EC dysfunction plays a pivotal role in the development of SSc vasculopathy, and the initial damage affecting these cells can be induced by various factors including environmental factors, viral infections, anti-endothelial autoantibodies, ischemia-reperfusion events, and reactive oxygen species [2,6,12,13]. Once injured, ECs can undergo different outcomes such as dysfunctional activation or apoptosis. When dysfunctionally activated, ECs are incapable of performing angiogenesis, and, releasing higher levels of endothelin-1 (ET-1) and lower levels of nitric oxide and prostacyclin, leads to an imbalance that finally results in vasospasm, intimal proliferation, and vascular wall fibrosis [2,6,12]. Furthermore, activated ECs also exhibit changes in their cytoskeleton, loss of tight junctions, and the increased expression of adhesion molecules and cytokines, all of which promote interactions with circulating inflammatory/immune cells and contribute to perivascular inflammation [2,6,12]. Additionally, these cells can cause platelet activation and intravascular fibrin deposition, ultimately leading to luminal narrowing, vessel occlusion, and tissue hypoxia [2,6,12]. Conversely, EC apoptosis results in the loss of peripheral microcirculation, leading to a chronic state of tissue ischemia that cannot be compensated by sufficient angiogenesis [2,6,12]. Besides EC activation and apoptosis, SSc defective angiogenesis and capillary loss can further result from EndoMT, a process during which ECs undergo a phenotypic change toward profibrotic myofibroblasts contributing to tissue fibrosis [12,14]. Indeed, a simultaneous expression of endothelial markers (CD31, von Willebrand factor, and vascular endothelial (VE)-cadherin) and myofibroblast markers (α-smooth muscle actin (α-SMA), S100A4/fibroblast specific protein-1, and type I collagen) has been observed in ECs from SSc skin and lungs, where this transdifferentiation may contribute to both SSc-related PAH and interstitial lung disease (ILD) [12,14]. Interestingly, SSc serum was demonstrated to exert a pro-EndoMT effect on healthy dermal microvascular ECs (MVECs), presumably through the MMP-12-dependent cleavage of urokinase-type plasminogen activator receptor (uPAR), which is also known to be involved in SSc defective angiogenesis and the fibroblast-to-myofibroblast transition [12,14]. Of note, even the downregulation of the transcription factor friend leukemia integration factor 1 (Fli1) has been implicated in SSc-related EndoMT [12,14]. Strikingly, the occurrence of EndoMT seems to be implicated in different morphological features of SSc vasculopathy, i.e., destructive and fibroproliferative vasculopathy [10,14]. Indeed, when EndoMT affects the capillary network, it results in microvessel rarefaction and concomitant perivascular fibrosis, while when occurring in small arteries/arterioles, it contributes to myofibroblast accumulation within the vessel wall, with consequent intima proliferation and occlusive vascular disease [10,14]. Notably, using spatial proteomics, a very recent study by Rius Rigau et al. [15] confirmed the presence of a CD34+/α-SMA+/CD31+ EC subpopulation that was increased in the skin of SSc patients and expressed high levels of different transcription factors known to promote EndoMT. Besides directly differentiating into profibrotic myofibroblasts, ECs have also been recently demonstrated to acquire a senescence-associated secretory phenotype characterized by the release of exosomes and different profibrotic/proinflammatory mediators, thus promoting fibrosis in an indirect way [12]. Senescent ECs are in a state of permanent cell cycle arrest and display morphological and metabolic changes including chromatin reorganization, and an altered gene expression associated with lower proliferation and defective angiogenic abilities [16,17]. In addition, senescent ECs also seem to preferentially undergo EndoMT [18]. In SSc, whole exome sequencing recently revealed the presence of numerous somatic mutations with a clock-like senescence signature in skin biopsies of patients suffering from severe skin and lung involvement [19], while gene expression meta-analysis in the lung of SSc patients with ILD demonstrated cellular senescence signatures in fibroblasts and epithelial cells, with a significant loss of ECs [20]. Interestingly, SSc sera containing anti-CENP-B and anti-TOPO-1 disease-specific autoantibodies were found to promote in vitro EC senescence independently from the p53–p21 pathway [21].

**Figure 1:**
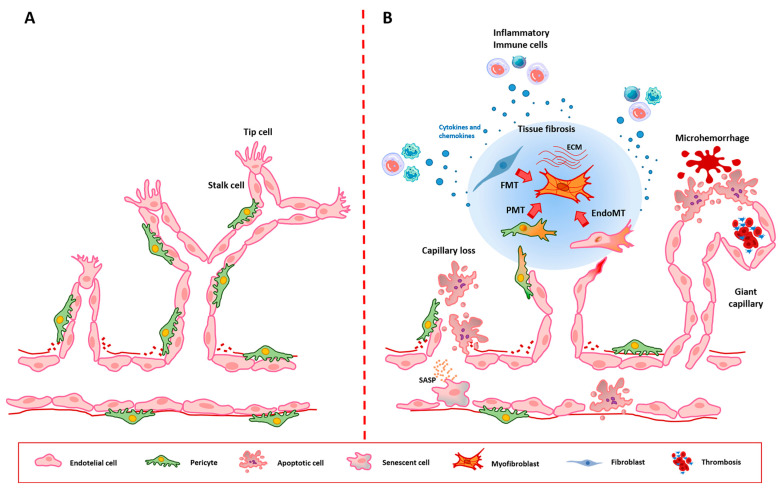
Schematic representation of normal sprouting angiogenesis (**A**) and SSc disturbed angiogenesis and related EC dysfunctional processes (**B**). (**A**) During normal sprouting angiogenesis, endothelial cells (ECs) sprout from a capillary to form a novel capillary network. The process starts with an increase in vascular permeability, with consequent extravasation of plasma proteins that act as a provisional scaffold for ECs. Once activated, specific ECs known as “tip” and “stalk” cells proliferate and migrate into the surrounding tissue by degrading the vascular basement membrane. The initially immature and leaky newly formed vessels undergo remodeling and stabilization through the interaction with pericytes. (**B**) In SSc disturbed sprouting angiogenesis, injured ECs mainly undergo dysfunctional activation or apoptosis clinically manifesting as microhemorrhages, giant capillaries, and capillary loss. When dysfunctionally activated, ECs are incapable of performing angiogenesis, and cause platelet activation and intravascular fibrin deposition (thrombosis), followed by luminal narrowing, vessel occlusion, and tissue hypoxia. EC apoptosis results in capillary rarefaction and consequent tissue ischemia. SSc defective angiogenesis can be further promoted by the endothelial-to-mesenchymal transition (EndoMT), a process during which ECs undergo a phenotypic change toward profibrotic myofibroblasts contributing to tissue fibrosis with abnormal deposition of extracellular matrix (ECM). Myofibroblasts can also originate from tissue-resident fibroblasts through a fibroblast-to-myofibroblast transition (FMT) and perivascular pericytes through a pericyte-to-myofibroblast transition (PMT). SSc ECs may also acquire a senescence-associated secretory phenotype (SASP) characterized by the release of exosomes and different profibrotic/proinflammatory mediators. Perivascular inflammatory/immune cells recruited by dysfunctional ECs release a plethora of cytokines and chemokines that contribute to defective angiogenesis and fibrosis.

A schematic comparative representation of normal sprouting angiogenesis vs. SSc disturbed angiogenesis and related EC dysfunctional processes is shown in Figure 1.

### 3.1. Most Recent Molecular Mechanisms Involved in Impaired Angiogenesis and EndoMT

Platelet factor 4, also known as CXCL4, is a chemokine that, once released by platelets upon endothelial injury, exerts anti-angiogenic properties by inhibiting both EC proliferation and migration. In a recent study, SSc patients with DUs and an early NVC pattern showed increased circulating levels of CXCL4 [22]. In the same study, the proliferation, migration, and tube formation capability of human umbilical vein ECs (HUVECs) were all significantly inhibited upon stimulation with SSc serum, an effect that was prevented by adding a CXCL4 neutralizing antibody [22]. From a molecular point of view, CXCL4 was found to contribute to SSc peripheral vasculopathy through Fli1 downregulation [22].

Another chemokine involved in angiogenesis is C-C motif chemokine ligand 20 (CCL20), which was found to induce the proliferation, invasion, and in vitro tube formation of different cancer cells through the interaction with its receptor CCR6 [23]. In SSc, higher serum CCL20 levels correlated with clinical parameters of PAH [24], and an increase in CCL20 expression paralleled by a decrease in CCR6 was reported in dermal SSc fibroblasts and MVECs, respectively [25]. Moreover, Fli1 deficiency was shown to augment CCR6 expression in healthy dermal MVECs [25]. As Fli1 deficiency has been demonstrated to promote the development of SSc vasculopathy, the authors suggested that CCR6 might be an additional factor contributing to this pathologic mechanism [25].

In another recent in vitro study, adipsin, an adipokine whose high circulating levels were previously associated with SSc-related PAH [26,27], was found to be augmented in the dermal small vessels of SSc-involved skin compared to those of healthy skin [27]. Notably, Fli1 silencing in human dermal MVECs was demonstrated to enhance both gene and protein expression of this adipokine, further suggesting that Fli1 deficiency may influence EC phenotype in SSc [27]. 

CD248 (also known as endosialin) is a type 1 membrane protein belonging to the C-type lectin family which, during angiogenesis, promotes EC interactions with fibroblasts and pericytes by binding to the endothelial-specific ECM glycoprotein multimerin-2 (MMRN-2), thus avoiding angiogenesis activation [28]. In SSc, an increased expression of CD248 and MMRN-2 was found in dermal fibroblasts and MVECs, respectively, with CD248+ SSc fibroblasts being able not only to inhibit the angiogenic performance of healthy MVECs but also induce their apoptosis, suggesting that increased CD248 may contribute to microvascular rarefaction [29].

In a study published by Henrot et al. in 2020, proangiogenic cellular communication network factor 3 (CCN3) expression was found to be significantly lowered in dermal microvessels of both SSc-affected skin and explanted SSc MVECs [30]. Moreover, CCN3 blockade in healthy MVECs resulted in the impairment of their migratory and angiogenic abilities, while treatment of SSc MVECs with recombinant human CCN3 significantly improved their in vitro angiogenesis [30].

In the same year, Li et al. studied the contribution of exosomes, i.e., cell-derived extracellular spherical vesicles containing DNAs, RNAs, and proteins and contributing to intercellular signaling, in SSc angiogenesis [31]. In particular, they found that exosomes isolated from the neutrophils of SSc patients were able to inhibit healthy dermal MVEC proliferation and migration, and that such an effect was mediated by the high levels of the calcium-binding proteins S100A8 and S100A9 that specifically bind to ECs [31].

The Epstein–Barr Virus (EBV) has been associated with different autoimmune disorders, including SSc, and since viral lytic antigens have been found in dermal vessels of SSc patients, ECs are supposed to be a possible EBV target in such a disease [32]. In this context, an increased EBV DNA load was reported in SSc circulation, B cells, and monocytes. In vitro, human monocytes infected with recombinant EBV were able to transmit the virus to ECs and activate markers of vascular injury, suggesting that monocytes may contribute to vascular damage in SSc [32].

In a study by Huang et al., growth factor receptor-bound protein-2 (GRB2), a signal transduction protein indispensable for several cellular functions, was found to be overexpressed in SSc ECs [33]. Of note, GRB2 downregulation not only resulted in a significant decrease in EC apoptosis both in an in vitro model of reactive oxygen species-mediated EC damage and in a bleomycin-treated mouse but also in the amelioration of skin fibrosis in the same models [33]. Thus, since GRB2 inhibition can prevent EC apoptosis and attenuate skin fibrosis, this molecule was proposed as a new therapeutic target [33].

In a quite innovative study, SSc angiogenesis was investigated with a 3D microvessel-on-a-chip on an OrganoPlate platform [34]. In this model, the authors replaced fetal bovine serum with SSc human serum and confirmed its antiangiogenic effects, thus paving the way for the assessment of new potential compounds able to protect from microvascular destabilization or regression in these disease-mimicking conditions [34].

A decreased expression of hemeoxygenase-1 (HO-1), a proangiogenic antioxidant enzyme, was reported in transforming growth factor (TGF)-β-treated SSc fibroblasts and HUVECs [35]. Interestingly, HUVECs cultured onto confluent dermal SSc fibroblasts and challenged with an inhibitor of HO-1 showed a significantly reduced ability to form tubules, suggesting that the downregulation of HO-1-mediated signaling could be associated with EC dysfunction and vasculopathy in SSc [35].

The contribution of oncostatin M (OSM), a member of the interleukin (IL)-6 family already found to be elevated in SSc circulation, and its receptor OSMRβ to SSc-related EndoMT has been recently investigated by Marden et al. [36]. In this study, OSMRβ expression was reported to be higher in dermal ECs from SSc patients compared to healthy controls, and treatment of healthy dermal MVECs with OSM was able to stimulate the expression of genes and proteins related to EndoMT via OSMRβ and STAT3 signaling pathways, suggesting that the activation of the OSM/OSMRβ axis in ECs could contribute to SSc pathogenesis [36].

EndoMT promotion and angiogenesis impairment have also been recently attributed to extracellular acidification, determined by the increased glycolytic metabolism of SSc dermal fibroblasts [37]. Indeed, in vitro stimulation with an SSc fibroblast-derived conditioned medium significantly decreased EC tube formation and invasion, and ECs exposed to experimentally induced extracellular acidosis were found to lose angiogenic capabilities and acquire a myofibroblast-like morphology and phenotype [37]. As a molecular link between extracellular acidosis and EC dysfunction, the authors found that acidic ECs were characterized by an upregulation of MMP-12, which in turn cleaves and inactivates uPAR in ECs [37]. Thus, extracellular acidosis caused by SSc fibroblasts was supposed to promote EC dysfunction through MMP-12-mediated uPAR cleavage, a mechanism already known for its implication in both SSc-related defective angiogenesis and EndoMT [37].

In a very recent study using differentiation trajectories at a single cell level, Ma et al. demonstrated that in SSc, EndoMT starts from a cluster of ECs expressing the *GJA4* gene, which encodes connexin37 and is characteristic of arteriolar ECs [38]. Moreover, the authors identified a significant role of Hippo pathway effectors in EndoMT and proposed that the modulation of Hippo signaling could be effective in reverting this key pathogenic process [38].

### 3.2. Defective Lymphangiogenesis and Occurrence of Lymphatic EndoMT

The lymphatic system mirrors the circulatory one and plays key roles in maintaining fluid homeostasis and organizing and modulating the immune system response [39]. Lymphatic microvessels initiate within tissues as blind-ended thin-walled capillaries that are constituted by lymphatic ECs and lack a basement membrane, thus allowing the passage of tissue interstitial fluid [39]. These initial lymphatic capillaries merge into larger collecting lymphatic vessels that, encircled by pericytes and smooth muscle cells, ultimately deliver the lymph into the bloodstream [39]. During lymphangiogenesis, new lymphatic vessels originate from preexisting ones through the sprouting of lymphatic MVECs that, once activated, proliferate and migrate into the ECM [39]. In such a well-coordinated process, VEGF-C is known to play a pivotal role by modulating lymphatic MVEC migration and proliferation through its interaction with the VEGF receptor-3 (VEGFR-3)/Flt-4 and neuropilin-2 (NRP-2) co-receptor [39]. As far as SSc is concerned, a severe reduction in the dermal microlymphatic network has been demonstrated in the clinically-affected skin of patients, and it has been hypothesized that dermal lymphatic microangiopathy may be involved in the early edematous phase of the disease, when painless “puffy fingers” are a clinical manifestation [6,40]. On these bases, our group has recently investigated the possible contribution of impaired lymphangiogenesis to SSc peripheral microvasculopathy [41,42,43]. In a first study, we found that SSc sera not only significantly inhibited the proliferation, invasion, wound healing capacity, and capillary morphogenesis of normal human dermal lymphatic MVECs, but also led to a significant downregulation of both VEGFR-3/Flt-4 and NRP-2 [41]. These data collectively demonstrated that the SSc pathologic microenvironment can deeply impair different lymphangiogenesis steps, presumably by downregulating the pro-lymphangiogenic VEGFR-3/NRP-2 signaling [41]. Successively, we demonstrated for the first time the occurrence of a lymphatic EndoMT process in SSc, with the lymphatic endothelium representing a novel source of profibrotic myofibroblasts [43]. Indeed, transitional lymphatic ECs expressing both of their specific markers, lymphatic vessel endothelial hyaluronan receptor-1 and α-SMA, were detected in SSc fibrotic skin, and human dermal lymphatic MVECs cultured in the presence of SSc sera were found to acquire a myofibroblast-like morphofunctional phenotype [43]. Interestingly, a very recent study performing single-cell RNA sequencing (scRNA-seq) and spatial-seq on SSc skin revealed that the lymphatic EC sub-cluster had a high ECM score owing to an increased expression of ECM pathway genes [38].

Besides lymphangiogenesis, lymphvasculogenesis, i.e., the formation of novel lymphatic vessels from circulating bone marrow-derived lymphatic endothelial progenitor cells (EPCs) differentiating into mature lymphatic ECs, may also contribute to SSc-related peripheral microvasculopathy. In fact, SSc patients with DUs were found not only to display lower levels of circulating lymphatic EPCs (defined as CD34+/CD133+/VEGFR-3+ cells), compared with both patients without DUs and healthy controls, but also lower levels of surface VEGFR-3 on such cells [42].

## 4. Circulating Factors and Cells Involved in SSc Defective Angiogenesis

Over the years, several circulating elements including proangiogenic molecules (e.g., VEGF, endoglin, and ET-1), antiangiogenic factors (e.g., VEGF165b, endostatin, and pentraxin-3), cell adhesion molecules, neurovascular guidance molecules, and sirtuins have been associated with SSc clinical features of impaired angiogenesis and proposed as vascular biomarkers [44,45]. Moreover, disease vasculopathy has been correlated with both the number of circulating angiogenic cells and the presence of specific autoantibodies [8,44].

### 4.1. Circulating Factors

Resistin is a hormone secreted by adipocytes and mononuclear cells, is able to promote angiogenesis, and has recently been discovered to be involved in microvascular dysfunction [46]. In a study in which circulating resistin levels were measured in SSc patients at baseline and after a 52-week follow-up, this adipokine was found to be higher in SSc patients compared to controls and, among patients, to be lower in those with an early NVC pattern [47]. After 52 weeks, serum resistin was significantly higher in patients with new DUs than in those without, with multivariate analyses demonstrating that it was associated with the development of new DUs, thus representing a new predictive marker of this clinical manifestation [47].

In another study by the same group, kynurenic acid, an anti-inflammatory and anti-oxidant molecule secreted by ECs in response to vascular inflammation, was measured in the circulation of SSc patients and correlated with both the NVC pattern and peripheral blood perfusion of hands, assessed by laser speckle contrast analysis and quantified as a proximal–distal gradient (PDG) [48]. Interestingly, PDG and serum kynurenic acid values were significantly lower in SSc patients with a late NVC pattern compared to those with early and active NVC, and patients without PDG, i.e., patients with high vascular damage, had significantly lower kynurenic acid values than those with PDG [48]. Based on these data, the authors suggested that this molecule may be associated with early endothelial dysfunction [48].

In recent years, Leleu et al. demonstrated that microparticles (vesicular structures deriving from different cellular sources known to mediate cell-cell communication) may also contribute to SSc vasculopathy, as shown by the evidence that they were significantly higher in SSc patients with active DUs [49].

Circulating levels of VEGF-C and the soluble form of its cognate receptor, soluble VEGFR-3, were measured in two well-characterized SSc cohorts and were found to be predictive of the development of PAH, suggesting that lymphangiogenesis is deregulated during PAH development [50].

Finally, specific circulating SSc autoantibodies embedded in immune complexes were found to exert proinflammatory and/or profibrotic effects on HUVECs by modulating the expression of several molecules involved in vascular impairment (ET-1 and IL-8), inflammation (intercellular adhesion molecule-1, IL-6), and fibrosis (TGF-β1) [51].

### 4.2. Circulating Angiogenic Cells

Bone marrow-derived CD34+ proangiogenic hematopoietic cells (PHCs) are multipotent circulating cells able to differentiate into several cell types, including smooth muscle cells and EPCs, thus contributing to vascular network remodeling [52]. Recently, the association among CD34+ PHCs, the EC-released proteoglycan endocan, and clinical parameters was assessed in SSc patients with PAH [53]. In this study, the number of circulating CD34+ cells was found to correlate with both endocan plasma levels and pulmonary arterial pressure values, leading the authors to hypothesize that endocan and CD34+ PHCs may represent eligible markers of disease status [53].

Over the years there has also been increasing interest in EPCs, the circulating cells responsible for postnatal vasculogenesis and identifiable by flow cytometry as CD34+, CD133+, and/or VEGFR-2+ [2]. Despite initial conflicting results about their numbers in SSc patients, presumably because of the different procedures used in different laboratories, the number of circulating EPCs has recently been shown to be reduced in SSc when employing more standardized protocols [2,54].

SSc-related peripheral vascular features have also been evaluated in relation to circulating angiogenic T cells (Tang), a specific T cell population found to induce new blood vessel formation and repair and characterized by the co-expression of CD3, CD31, and CXCR4 (i.e., the receptor for the CXC chemokine stromal cell-derived factor-1 (SDF-1)/CXCL12) [55,56]. In a first study, Tang cells were found to be significantly higher in SSc patients with DUs compared to those without, and in patients with late NVC patterns compared to those with early/active NVC [55]. Moreover, their percentage in SSc peripheral blood positively correlated with the levels of VEGF and MMP-9, and inversely correlated with those of SDF-1 and EPCs [55]. In SSc skin, Tang cells were often detected within perivascular inflammatory infiltrates [55]. Interestingly, in a more recent study dividing Tang cells into two different subgroups according to the expression of CD4 or CD8, it was demonstrated that the increased number of total Tang cells in SSc patients was mostly due to a higher rate of CD8+ Tang cells, indicating that this subpopulation may have a more critical pathologic effect [56]. In addition, the percentage of Tang cells was found to be higher in patients with PAH [56]. Collectively, these studies suggest that Tang cells may be potential biomarkers of peripheral vascular damage severity in SSc [55,56].

Circulating ECs (CECs), rare in healthy subjects, are mature ECs that, detached from the basement membrane after vessel wall injury, have been detected in a wide range of diseases characterized by vascular perturbation. In a recent study using a highly standardized flow cytometry method, the number of CECs was confirmed to be extremely low in healthy controls compared to SSc and, among patients, to be higher in those with DUs and late NVC patterns [57]. When measuring CECs 12 months after the first assessment, the authors found a correlation between their number and the clinical worsening of patients, indicating that CECs might be employed as a reliable marker of disease severity [57].

Since CECs and endothelial microparticles, i.e., microvesicles released from the cellular membrane during EC activation or apoptosis, both reflect the degree of endothelial injury, a recent study investigated their possible relationship with anti-EC antibodies (AECAs) and assessed if AECAs may contribute to endothelial damage by evaluating the number of EPCs [58]. In particular, the authors found that AECAs correlated with increased levels of total and apoptotic endothelial microparticles and that patients with AECAs were characterized by a higher number of CECs. Moreover, as AECAs are negatively associated with angiogenic EPCs, these autoantibodies were suggested to play a role in the impairment of vascular repair [58].

## 5. Genetic and Epigenetic Mechanisms Involved in SSc Defective Angiogenesis

Genetic modifications are known to play a significant role in SSc pathogenesis, but taken alone they do not allow clarification of the occurrence of the disease [59,60]. Indeed, SSc is thought to be determined by complex interactions between genetic predispositions and epigenetic modifications such as DNA methylation, histone modifications, and microRNAs (miRNAs), which are able to regulate gene expression without altering the DNA sequence [61,62,63]. In the last two decades, besides the known contribution of human leukocyte antigen (HLA) class II haplotypes to the risk of developing SSc, various multicenter studies discovered several non-HLA loci contributing to SSc pathogenesis, but very few pointed toward potential genetic modifications relevant for disease vasculopathy [59,60]. In 2020, Takagi et al. genotyped four single nucleotide polymorphisms of the *HIF1A* gene in a Japanese SSc population and found that the AA genotype at rs12434438 was not only significantly increased in SSc patients with PAH compared to those without but also associated with the severity of this pulmonary condition [64].

In a genome-wide association study performed on a Han Chinese population, the rs4317244 single nucleotide polymorphism located upstream of the LRP2 binding protein (*LRP2BP*) gene, was found to associate with SSc, with the alternative G allele significantly correlating with a decreased expression of *LRP2BP* in arteries [65]. Moreover, as an in vitro *LRP2BP* knockdown in lung and skin MVECs was demonstrated to impair tight junction integrity, inhibit morphogenesis, and promote apoptosis, the authors suggested that the alternative G allele of rs4317244 might contribute to endothelial injury in SSc through *LRP2BP* downregulation in ECs [65].

In a recent study evaluating chromatin accessibility patterns and transcription factor footprints in SSc MVECs, chromatin availability was found to be significantly reduced in SSc patients compared to healthy controls, particularly at the level of neuronal genes including *NRXN1*, while the chromatin binding of the ETV2 transcription factor (essential for vascular development) was found to be increased [66]. Functional experiments on human dermal MVECs demonstrated that an *NRXN1* knockdown significantly reduced in vitro angiogenesis, while an *ETV2* knockdown exerted opposite effects [66]. Interestingly, the expression of *CDKN1A* (gene encoding for p21) was significantly augmented in SSc MVECs, suggesting the presence of a senescent phenotype [66]. 

Different miRNAs identified in the blood or tissues of SSc patients have been implicated over the years in SSc defective angiogenesis and EC dysfunction and have been exhaustively discussed in previous reviews [67,68]. Amongst the most recent studies, the expression of EC-specific miRNA-126, located within the EGF-like domain-containing protein-7 (*EGFL7*) gene, and known to block VEGF-negative regulators such as the 9prout-related protein-1 (SPRED1) and phosphoinositide-3 kinase regulatory subunit-2 (PIK3R2), has been evaluated in ECs from SSc patients [69]. In particular, downregulation of miRNA-126 and its host gene and concomitant upregulation of *SPRED1* and *PIK3R2* were found in SSc MVECs, leading to a reduced VEGF-dependent angiogenic response in such cells [69]. Heavy methylation was also reported in the miRNA-126 promoter region in SSc MVECs, suggesting that proangiogenic miRNA-126 administration or epigenetic regulation of its expression might potentially endorse angiogenesis in SSc [69].

In another study assessing the genome-wide DNA methylation signature in SSc and healthy MVECs, it was found that in the former, differentially hypermethylated genes were involved in angiogenesis, while hypomethylated genes were involved in vascular smooth muscle contraction and adherens junctions [70].

Finally, when investigating the effect of miRNA-30c on the pathogenesis of SSc, Kanno et al. demonstrated that this miRNA was able to prevent vascular dysfunction in the skin of bleomycin-treated mice, thus exerting anti-angiopathy effects [71].

## 6. Transcriptomic and Proteomic Evidence for SSc Endothelial Cell Dysfunction

RNA microarray analysis was performed to study global gene expression differences between lung MVECs explanted from SSc patients with ILD and normal cells [72]. As a result, several genes related to interferon (IFN) pathways, antiviral responses, and EndoMT were found to be overexpressed in SSc-ILD pulmonary MVECs, suggesting that EC injury determined by viral infections may initiate and perpetuate vasculopathy in SSc-ILD [72].

Similarly, through scRNA-seq, IFN-1 signature was reported not only to be significantly prominent in dermal SSc MVECs compared to controls, but also to be statistically different in the two SSc skin subtypes, being indeed higher in patients with diffuse cutaneous SSc vs. those with limited cutaneous SSc [73].

In another study, transcriptome analysis and scRNA-seq of SSc skin biopsies allowed the identification of downregulation of the sperm-associated antigen-17 (*SPAG17*) gene, especially in ECs and fibroblasts [74]. Interestingly, *SPAG17* knockdown in human MVECs was accompanied by the acquisition of a myofibroblast-like phenotype and increased sensitivity to profibrotic stimuli [74]. Through ATAC-seq analysis, the authors finally demonstrated that reduced *SPAG17* levels in SSc MVECs were caused by reduced chromatin accessibility at the *SPAG17* locus [74]. RNA-sequencing and gene set enrichment analysis on SSc skin biopsies also revealed that tissues were enriched in signatures associated with keratinization, ECM generation, and negative regulation of angiogenesis [75,76].

In another very recent study on SSc skin, scRNA-seq revealed an abnormal upregulation of two previously unidentified SSc EC subpopulations, namely tip ECs and proliferating ECs, suggesting the presence of proangiogenic activities in compensation for vascular injury [77]. Moreover, open chromatin profiling integrated with transcriptomics unraveled higher chromatin accessibility for the ETS family transcription factors in SSc ECs, indicating a possible regulatory role of such transcriptional factors in driving dysregulated EC phenotypes [77].

Finally, scRNA-seq profiles of pulmonary ECs from patients with SSc-associated PAH identified three disease-associated EC populations, i.e., two angiogenic EC subtypes represented by tip ECs and the more quiescent/stabilized phalanx ECs, and ECs in transition toward a myofibroblast-like phenotype [78].

Proteomic profiles were recently assessed, via a discovery/validation approach, in serum samples of preclinical SSc patients defined as subjects presenting with Raynaud’s phenomenon, positive NVC, and SSc-specific antibodies without any other sign of definite disease [79]. To determine the progression to definite SSc, prospective data were also obtained after 5 years [79]. In this study, preclinical SSc patients displayed a distinct protein profile, with proteins associated with EC injury, vasculopathy, and collagen turnover characterizing the progression from a preclinical disease stage to a definite one [79]. In particular, endostatin, a basic fibroblast growth factor, and the platelet-activating factor acetylhydrolase-β subunit proteins, involved in both angiogenesis and fibrosis regulation, emerged as the most strongly associated with SSc progression [79].

A multi-tier study including an aptamer-based proteomic analysis and a validation on independent cohorts identified several serum proteins involved in ECM formation, angiogenesis, and vascular remodeling that not only allowed the discrimination of SSc patients from healthy subjects, but also associated them with disease duration and clinical manifestations such as ILD, skin involvement, and autoantibody profile [80]. 

## 7. Animal Models of SSc Vasculopathy

All the animal models developed for SSc display a fibrotic phenotype, but only a few of them are able to reproduce EC dysfunction and defective angiogenesis [81].

### 7.1. Endothelial Fli1-Deficient Mouse and Bleomycin-Treated Fli1-Haploinsufficient (+/−) Mouse

Mice with the conditional *Fli1* gene removal in ECs were found not only to develop disorganized dermal vascular networks, with a significant impairment of vessel integrity and a strong increase in vessel permeability but also exhibited an important downregulation of EC markers, all vascular abnormalities that can be detected in SSc human microvasculature [82]. Similarly, *Fli1* haploinsufficiency in bleomycin-treated mice has been found to disturb angiogenesis and induce a profibrotic phenotype in dermal ECs by facilitating bleomycin-induced EndoMT [83]. Indeed, bleomycin-treated *Fli*1+/− mice presented with a higher number of fibroblast-specific protein-1/VE-cadherin double-positive cells in the lower dermis compared to bleomycin-treated wild-type mice [83].

### 7.2. Klf5 and Fli1 Gene Double Heterozygous (Klf5+/−;Fli1+/−) Mouse

Mice with a double heterozygous deficiency of Krüppel-like factor-5 (*Klf5*) and *Fli1* genes represent an SSc animal model able to recapitulate all the three main pathological features of the disease in their specific chronological order, i.e., autoimmunity, microvasculopathy, and tissue fibrosis [84]. Such an experimental model has been recently used to assess neovascularization and angiogenesis using an in vivo Matrigel plug assay and in vitro tube formation assay [85]. As a result, the authors found that neovascularization was reduced in skin-embedded Matrigel plugs and that MVECs isolated from the derma of *Klf5*+/−; *Fli1*+/− mice were unable to perform in vitro angiogenesis and displayed a lower expression of the EC markers VE-cadherin and CD31, suggesting the occurrence of an EndoMT process [85].

### 7.3. Fra-2 Transgenic Mouse

Mice with an ectopic expression of the transcription factor Fos-related antigen-2 (Fra-2) manifest several features of SSc vasculopathy, as they display a significant loss of small blood vessels, with a decrease in dermal capillary density and an increase in early EC apoptosis [86]. Moreover, these animals have been reported to develop proliferative vasculopathy in the lungs closely resembling SSc-related PAH [87]. Interestingly, treatment of *Fra-2* transgenic mice with the antifibrotic compound nintedanib, currently approved for the management of SSc-associated ILD, was found to reduce dermal MVEC apoptosis and ameliorate capillary loss, all effects that were associated with the normalization of circulating VEGF [88]. In another recent study comparing the effects of nintedanib and mycophenolate mofetil, only nintedanib was found to significantly ameliorate pulmonary vascular remodeling, as well as reduce dermal MVEC apoptosis [89]. Conversely, pirfenidone, another antifibrotic compound with therapeutic efficacy in ILD, was found to aggravate vascular remodeling in *Fra-2* transgenic mice by inducing a strong downregulation of VE-cadherin in lung ECs [90]. Finally, adenosine depletion with PEGylated adenosine deaminase was found to inhibit skin MVEC apoptosis and dampen capillary rarefaction in *Fra-2* transgenic mice [91].

### 7.4. uPAR-Deficient Mouse

As previously mentioned, uPAR cleavage/inactivation has been implicated in SSc peripheral microvasculopathy (i.e., defective angiogenesis and EndoMT) and the fibroblast-to-myofibroblast transition [12,14]. On these bases, Manetti et al. investigated whether a *uPAR* gene knockout in mice could mimic human SSc by determining both tissue fibrosis and peripheral microvasculopathy [92]. As a result, the skin of *uPAR*-deficient mice was found not only to develop progressive dermal fibrosis but also to be characterized by significant EC apoptosis and severe capillary reduction [92]. Successively, the presence of transitional EndoMT cells was found in the dermal microvessels of these mice [93].

### 7.5. Transgenic Mouse with Endothelial Cell-Specific Inducible Expression of Constitutively Active TGF-β Receptor I

This transgenic model was obtained by interbreeding animals characterized by a constitutively active TGF-β receptor I with mice containing the EC-specific *Cdh5* gene promoter directing the tamoxifen-inducible expression of the Cre-ERT2 cassette so that tamoxifen administration results in a constitutive TGF-β signaling activation limited to ECs [94]. This mouse model was found to develop skin and pulmonary fibrosis, as well as the induction of EndoMT in pulmonary vessels [94].

### 7.6. Tumor Necrosis Factor-Transgenic Mouse Model

Tumor necrosis factor (*TNF*)-transgenic mice carry a modified human *TNF* transgene in which the 3′-region of the *TNF* gene has been replaced with that of the human *β-globin* gene [81,95]. The *TNF*-transgenic mice have been proposed as a novel animal model of SSc-PAH, as they display a pulmonary vasculopathy characterized by collagen deposition in the wall of small arteries/arterioles and vascular occlusion, and their lungs show signs of EndoMT and dysregulated angiogenic pathways [81,95].

### 7.7. Sclerodermatous Graft-Versus-Host Disease Mouse Model

This animal model, obtained by transplanting spleen cells from B10.D2 donor mice into lethally irradiated Rag-2 KO Balb/c mice, was found to display fibroproliferative vasculopathy, with ET-1 up-regulation in the perivascular area and a reduction in the vascular lumen [81,96].

### 7.8. Other Animal Models with SSc-like Pathological Vascular Features

In the SSc murine model obtained by subcutaneously immunizing animals with type V collagen emulsified in complete Freund adjuvant, the most important vascular manifestations were represented by EC activation and apoptosis, as demonstrated by an augmented expression of VEGF, ET-1, and caspase-3 [97].

Finally, adult Wistar rats subcutaneously injected with NaClO were found to undergo significant histopathological changes in lung microvasculature, with capillary disruption, narrowing of the lumen, and perivascular infiltration [98]. From an ultrastructural point of view, the animals presented several changes in lung ECs, including swollen mitochondria, fragmentation of the membranes of the granular endoplasmic reticulum, uneven thickening of the basement membrane, and numerous micro pinocytotic vesicles and vacuoles [98].

Although the abovementioned mouse models display some characteristics of SSc vasculopathy, it should be pointed out that most of them were not specifically developed and used to study this pathological aspect of the disease. Interestingly, an animal model resembling Raynaud’s phenomenon and displaying SSc-like nailfold capillary abnormalities has been recently proposed [99], hopefully encouraging the development of newer experimental models more specifically representing the pathologic vascular features of SSc.

A list of the most recent animal models manifesting SSc-like vascular manifestations is shown in Table 1.

## 8. Potential Therapeutic Approaches to Improve Angiogenesis and Reduce EndoMT in SSc

Iloprost is a prostacyclin analog commonly employed for the treatment of SSc patients featuring DUs and PAH [100,101]. In particular, it exerts vasodilatory properties by stimulating prostaglandin receptors on smooth muscle cells and ECs, thus activating adenylate cyclase and leading to the production of cyclic AMP. In addition, it seems to increase EPC turnover, decrease vascular permeability, and exert antioxidant effects [100,101]. In this context, sera of SSc patients treated with iloprost were found to reduce reactive oxygen species production, collagen synthesis, and apoptosis of human pulmonary MVECs compared to sera from untreated patients [102]. Moreover, Tsou et al. assessed the effect of iloprost on SSc dermal MVECs and found that this compound was able to restore junctional levels of VE-cadherin, thus reducing vascular permeability, increasing angiogenesis, and blocking EndoMT [103]. Finally, in a more recent study evaluating the effect of iloprost on the release of EC-derived extracellular vesicles as markers of endothelial dysfunction, iloprost administration to SSc patients was reported to affect EC vesicle release rate over time, thus showing potential long-term benefits [104].

Two different compounds, namely the antimalarial drug dihydroartemisinin and the phytochemical extract tanshinone IIA, were demonstrated to counteract EndoMT both in vitro in TGF-β1- or bleomycin-treated HUVECs and in vivo in the bleomycin-induced mouse model of skin fibrosis [105,106]. Interestingly, tanshinone IIA was also able to increase tube formation in bleomycin-treated HUVECs [106].

Three recent preliminary clinical studies suggested the efficacy of targeting IL-17 and IL-23 to improve clinical signs of SSc vasculopathy [107,108,109]. Indeed, treatment with monoclonal antibodies against IL-17A or IL-17A receptors was shown to reduce the occurrence of DUs and ameliorate skin fibrosis, while selective IL-23 inhibition with guselkumab resulted in a significant improvement in SSc NVC patterns [107,108,109].

Considering that soluble guanylate cyclase (sGC) stimulation has been proven to exert antifibrotic properties, in very recent work from our group dermal SSc MVECs were treated with the sGC stimulator MK-2947 to unravel if this compound could modulate their angiogenic abilities and myofibroblast-like phenotype [110]. Interestingly, MK-2947 treatment was able not only to improve cell proliferation, wound healing capacity, and in vitro angiogenesis but also dampened SSc MVEC profibrotic features through a reduction of phosphorylated-extracellular signal-regulated kinases 1 and 2 protein levels [110].

Given the beneficial effects of autologous hematopoietic stem cell transplantation (AHSCT) in SSc patients, NVC patterns and skin and serum vascular biomarkers were retrospectively assessed before and after AHSCT to investigate whether it may affect SSc vasculopathy [111]. One year after transplantation, NVC showed an increase in the number of capillaries with a decrease in the giant ones, while in skin biopsies only a decrease in E-selectin and an increase in angiopoietin-1 were detected [111]. As far as serum vascular markers are concerned, no changes were observed after AHSCT [111]. On these bases, the authors suggested that AHSCT may improve skin microvasculopathy but not influence circulating vascular markers [111].

Finally, as new autologous adipose-derived stem/stromal cell (ASC)-based therapies are currently under investigation for SSc, in a study from Velier et al., ASCs from SSc patients were co-cultured with healthy dermal MVECs in order to evaluate their paracrine angiogenic potential [112]. Interestingly, although to a lesser extent compared to healthy ASCs, SSc ASCs maintained the capacity to promote angiogenesis through paracrine mechanisms, further supporting a possible therapeutic use of these cells [112].

The most recent potential therapeutic approaches able to ameliorate SSc endothelial dysfunction are summarized in Table 2.

## 9. Conclusions

In SSc, endothelial dysfunction and defective angiogenesis, clinically manifesting with abnormal capillary architecture, progressive capillary reduction, and microcirculatory impairment mainly, are the consequences of several cellular and molecular mechanisms affecting ECs, including apoptosis, transdifferentiation into profibrotic myofibroblasts (i.e., EndoMT), or the acquisition of a senescence-associated secretory phenotype. Since considerable evidence exists that EC dysfunction with the impairment of compensatory angiogenesis is a primary pathogenetic step occurring before the onset of tissue fibrosis, a deeper unraveling of the underlying mechanisms is needed to develop new therapeutic strategies capable of treating SSc at early stages and/or block the progression from preclinical to established disease. In this scenario, the advent of omics technologies and the establishment of new in vitro tools and animal models we have witnessed in the past few years promise significant advancements toward precision medicine for SSc vasculopathy.

## Figures and Tables

**Table 1:** Animal models of SSc vasculopathy.

Animal Model	Vascular Manifestations	References
Endothelial *Fli1*-Deficient Mouse	Disorganized dermal vascular networks; loss of vascular integrity; downregulation of EC markers	[82]
Bleomycin-Treated *Fli1*-Haploinsufficient (+/−) Mouse	Disturbed angiogenesis; EndoMT in dermal vessels	[83]
*Klf5*+/−;*Fli1*+/− Mouse	Disturbed angiogenesis; EndoMT in dermal vessels	[85]
*Fra-2* Transgenic Mouse	Loss of small blood vessels; early EC apoptosis; proliferative vasculopathy in the lungs resembling SSc-related PAH	[86,87]
*uPAR*-Deficient Mouse	Dermal EC apoptosis and severe capillary reduction; EndoMT in dermal vessels	[92,93]
Transgenic Mouse with EC-Specific Inducible Expression of Constitutively Active TGF-βRI	EndoMT in pulmonary vessels	[94]
Type V Collagen Immunized Mouse	EC activation and apoptosis	[97]
Rats Subcutaneously Injected with NaClO	Capillary disruption, narrowing of vascular lumen, and perivascular infiltration in lungs	[98]
*TNF*-Transgenic Mouse	Pulmonary vasculopathy; EndoMT; dysregulated angiogenic pathways	[81,95]
Sclerodermatous Graft-Versus-Host Disease Mouse	Fibroproliferative vasculopathy	[81,96]

EC, endothelial cell; EndoMT, endothelial-to-mesenchymal transition; PAH, pulmonary arterial hypertension; SSc, systemic sclerosis.

**Table 2:** Therapeutic Approaches to Improve Angiogenesis and Reduce EndoMT in SSc.

Compound/Treatment	Mechanism of Action and Effects	References
Iloprost	Prostacyclin analog that increases EPC turnover, exerts antioxidant effects, reduces vascular permeability, increases angiogenesis, blocks EndoMT, and affects the release of EC-derived extracellular vesicles	[102,103,104]
Dihydroartemisinin	Antimalarial drug that counteracts EndoMT in vivo and in vitro	[105]
Tanshinone IIA	Phytochemical extract that counteracts EndoMT and increases tube formation in bleomycin-treated HUVECs	[106]
MK-2947	sGC stimulator that improves SSc MVEC proliferation, wound healing capacity and in vitro angiogenesis, and dampens SSc MVEC profibrotic features	[110]
Autologous hematopoietic stem cell transplantation	Increased number of capillaries, with a decrease in the giant ones; decreased E-selectin and increased angiopoietin-1 in skin biopsies	[111]
Autologous adipose-derived stem/stromal cells	Promotion of angiogenesis of dermal MVECs through paracrine mechanisms	[112]

EC, endothelial cell; EndoMT, endothelial-to-mesenchymal transition; EPC, endothelial progenitor cell; HUVECs, human umbilical vein endothelial cells; MVECs, microvascular endothelial cells; sGC, soluble guanylate cyclase; SSc, systemic sclerosis.
